# Observer efficiency in free-localization tasks with correlated noise

**DOI:** 10.3389/fpsyg.2014.00345

**Published:** 2014-05-01

**Authors:** Craig K. Abbey, Miguel P. Eckstein

**Affiliations:** Department of Psychological and Brain Sciences, University of CaliforniaSanta Barbara, CA, USA

**Keywords:** free-localization tasks, ideal observer theory, power-law noise, observer efficiency, image statistics

## Abstract

The efficiency of visual tasks involving localization has traditionally been evaluated using forced choice experiments that capitalize on independence across locations to simplify the performance of the ideal observer. However, developments in ideal observer analysis have shown how an ideal observer can be defined for free-localization tasks, where a target can appear anywhere in a defined search region and subjects respond by localizing the target. Since these tasks are representative of many real-world search tasks, it is of interest to evaluate the efficiency of observer performance in them. The central question of this work is whether humans are able to effectively use the information in a free-localization task relative to a similar task where target location is fixed. We use a yes-no detection task at a cued location as the reference for this comparison. Each of the tasks is evaluated using a Gaussian target profile embedded in four different Gaussian noise backgrounds having power-law noise power spectra with exponents ranging from 0 to 3. The free localization task had a square 6.7° search region. We report on two follow-up studies investigating efficiency in a detect-and-localize task, and the effect of processing the white-noise backgrounds. In the fixed-location detection task, we find average observer efficiency ranges from 35 to 59% for the different noise backgrounds. Observer efficiency improves dramatically in the tasks involving localization, ranging from 63 to 82% in the forced localization tasks and from 78 to 92% in the detect-and- localize tasks. Performance in white noise, the lowest efficiency condition, was improved by filtering to give them a power-law exponent of 2. Classification images, used to examine spatial frequency weights for the tasks, show better tuning to ideal weights in the free-localization tasks. The high absolute levels of efficiency suggest that observers are well-adapted to free-localization tasks.

## Introduction

The concept of calculation efficiency, which we refer to simply as efficiency, in the presence of image noise has been used extensively as a method for understanding visual processing since its seminal introduction by Barlow (Barlow, 1977, 1978; Barlow and Reeves, 1979). At the core of this measure is comparison with an optimal decision maker, the ideal observer, for a given task. The use of the ideal observer as yardstick for human performance implicitly controls for the relevant information present in stimuli used to perform a task. This topic has a long history in vision science, as well as areas of applied vision such as medical imaging. In the realm of vision science, there are many examples where efficiency is used to reveal the presence (or absence) of limitations and constraints in visual processing (Barlow, 1978; Barlow and Reeves, 1979; Burgess et al., 1981; Pelli, 1985; Legge et al., 1987; Geisler, 1989; Tjan et al., 1995). In imaging applications, efficiency is used to identify opportunities for image processing or other methodological changes that lead to improved performance in visual tasks (Myers et al., 1985; Wagner and Brown, 1985; Insana and Hall, 1994; Siewerdsen and Jaffray, 2000; Abbey et al., 2006).

Studies evaluating efficiency have often relied on experimental paradigms where the location of a target, if it is present, is explicitly defined through the use of location cues. Forced-choice paradigms, with two or more specified locations that serve as possible target locations, are a common choice (Burgess and Ghandeharian, 1984). These studies do involve spatial (or temporal) search, but it is a limited search that is confined to choosing between distinct, cued locations. The use of independent noise masking the target at each location makes the computation of the ideal observer considerably easier. Studies that have analyzed the ideal observer in tasks with location uncertainty on a quasi-continuous scale (i.e., limited to the pixelation of the stimulus) have generally utilized a detection or discrimination response that did not involve localizing the target (Park et al., 2005; Tjan and Nandy, 2006; Neri, 2010).

However, recent analysis by Khurd and Gindi (2005) have demonstrated how an ideal observer may be evaluated when targets can be located anywhere within a search region, and the task requires localizing targets to within a fixed distance, or more general acceptance region. This general paradigm has been used previously in medical imaging studies (Burgess et al., 2001; Bochud et al., 2004) due to the similarity with many clinical tasks that require identifying a location in the body for further assessment. However, these studies did not have the benefit of an ideal observer. The Khurd and Gindi approach leads to the definition of an optimal decision function, from which ideal observer performance can be extracted via simulation studies, as we do below. Extensions to the theory (Khurd et al., 2010) include methods for evaluating the presence of multiple targets that are beyond the scope of this work. There has been some use of this analysis to evaluate the role of regularization in emission computed tomography (Liu et al., 2009). However, we are not aware of any use of the ideal observer for examining efficiency in more general free-localization tasks.

**Figure 1:**
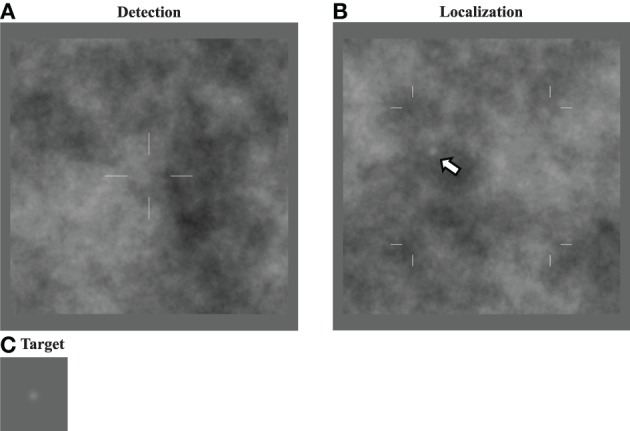
**Detection and localization stimuli**. Image displays for the detection **(A)** and localization **(B)** tasks. The target to be detected is a Gaussian (“bump”) profile**(C)** embedded in power-law noise with an exponent of 2 here. For the detection task, the target is located at the center of the cross when it is present. In the localization task, the target can be located anywhere within the search area indicated by the marks (arrow).

The main focus of this work is a comparison of fixed-location detection tasks—where a single target location is well cued—to free-localization tasks, where the subject must indicate the location of a target that can be anywhere in a defined search region. Figure 1 gives examples of the stimulus displays for each task. For the detection tasks, subjects render a decision on whether a Gaussian “bump” target profile is present or not at the cued location. In the localization tasks, the subject is required to indicate the location of the target profile, which will always be present somewhere in the search region. We are interested in comparing the efficiency of human observers in these two tasks, and understanding the mechanisms that can explain differences.

**Figure 2:**
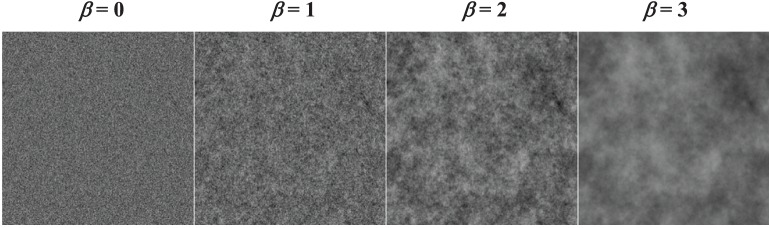
**Sample power-law textures**. Noisy backgrounds with different power-law exponents (β) are shown from the same underlying random number seed.

We are also interested in the role of the background image statistics on this process. For this reason, we evaluate four different Gaussian image textures. These are defined by their power spectra, which are constrained to be a power-law parameterized by the power-law exponent β. We evaluated four different background textures, based on β-values ranging from 0 (white noise) to 3 (See Figure 2 below). Natural scenes are often modeled as power-law processes with exponents that vary around β = 2 (Burton and Moorhead, 1987; Field, 1987). Various forms of x-ray images, for breast imaging in particular, have also been modeled as power-law processes with exponents ranging from less than 2 for computed tomography reconstructions (Metheany et al., 2008; Chen et al., 2012, 2013) to 3 or more for tomosynthesis (Engstrom et al., 2009) or projection images (Bochud et al., 1999; Burgess et al., 2001).

In addition to the main comparison of detection and localization tasks, two smaller follow-up studies were conducted to give additional insight on issues that arose from the primary study. One issue was the different nature of the two tasks, given that a yes-no type detection task requires maintaining some sort of detection criterion from trial to trial for choosing the response. The free-localization tasks do not require this. To investigate the effect of a detection criterion on free-localization tasks, we evaluated a detect-and localize (D&L) task, in which the target profile appeared at a random location in the search region in 50% of the trials, and was not present in the other 50% if the trials. As an alternative to indicating the target location, the subjects could also respond “not present” in these experiments. In this way, the requirement of maintaining a task criterion in the detection task was matched in a task with substantial spatial uncertainty.

The second follow-up study concerned the white-noise (β = 0) background condition, where lower efficiency than other power-law backgrounds was observed in both detection and localization tasks. In this case, we were interested in whether processing the images to have more favorable background statistics could improve performance. We evaluated task performance after filtering these images to have a power-law power spectrum with β = 2, which also modified the profile of the target.

## Methods

A total of 5 subjects participated in the primary comparison of efficiency between detection and forced-localization tasks. On subject (S1) was a coauthor of this work, and the other 4 were naïve to the purposes of the research and compensated for their participation. Of these, 3 subjects (S2, S4, and S5) participated in the secondary detect-and-localize experiments, and 3 subjects (S3, S4, and S5) participated in the secondary image-processing experiments.

### Stimulus and display properties

A monochrome CRT display (Imaging Systems, Minnetonka, Minnesota) with a dedicated controller (DOME, NDS Inc., San Jose, CA), was used for all experiments, which were conducted in a darkened room. The monitor was photometer-calibrated to an 8-bit linear lookup table (LUT) that ranged from 0.02–40 Cd/m^2^. Viewing distance of the subjects was not constrained. Subjects had normal vision or wore corrective lenses. After becoming familiar with the display procedure, and completing several sessions of experiments, measurements of each subject's comfortable viewing distance were made. The average viewing distance used was 64 cm, with a range of 51–70 cm. This average distance was used for all subsequent calculations of visual angle. The stimuli were generated as 256 by 256 pixel images, and these were magnified by a factor of two for display, making the effective pixel size 0.052° (0.583 mm).

The experiments used a Gaussain “bump” as a target added to stationary noise with a power-law power spectrum. The spatial standard deviation of the target was 3 pixels giving the displayed target a FWHM of 0.37°. A mean background level of 100 gray levels (gl) was added as well and the noise was scaled to have a pixel variance of 400 gl^2^, which is equivalent to a 20% RMS contrast on the linearized display that was used for the psychophysical studies. Target contrast varied over the different experiments, as described below.

Noise backgrounds were generated by filtering white noise to achieve a power-law power spectrum in the spatial-frequency domain, *S*_β_(*f*) = *C*_β_/*f*^β^, which we will identify by the power-law exponent, β. To avoid the singularity at *f* = 0, the DC component is set to the value of the first harmonic. The normalization constant, *C*_β_, is set so that the RMS contrast of the background is fixed at 20%. The noise generation filter for each background condition was set to be the square-root of *S*_β_(*f*). Examples of the different noise textures for the four values of β that we used are seen in Figure 2.

For detection tasks using the different backgrounds, targets had a 50% probability of being present in any given trial. Subjects were informed that this was the target probability. When present, the target was always located in the center of the image with the location indicated by cross-hairs, as shown in Figure 1. The observer response was obtained by capturing a mouse click outside the image area. Feedback (correct/incorrect) was given after each trial. While separate performance measures were determined for target present and target absent images (hit rate and false alarm rate), for purposes of fitting psychometric functions the proportion of correct responses was also used.

For localization tasks, the target was randomly located in the central region of the image, with borders delineated by hash marks. Subjects were informed that this was the search region. The central region consisted of 128 by 128 pixels (6.7 by 6.7°), and thus constituted one quarter of the total image area. The large border region was chosen to minimize any edge effects as well as effects from “wrap-around” from the filtering operation. Observers responded by clicking with a mouse on their selected location. Mouse-clicks that were 5 pixels (0.26°) or less from the center of the target were considered “correct,” and subject performance was measured as the proportion of correct responses. Subjects received feedback (correct/incorrect and true target location) after each trial.

Slight modifications to the experimental protocol above were used in the two follow-up studies. For the D&L tasks, there was a 50% probability that the target was present somewhere in the search region. Subjects responded with a mouse-click on the target location to indicate target presence at that location, or by a mouse-click outside the image to indicate target not present. Subjects received feedback (correct/incorrect and target location if applicable) after each trial.

**Figure 3:**
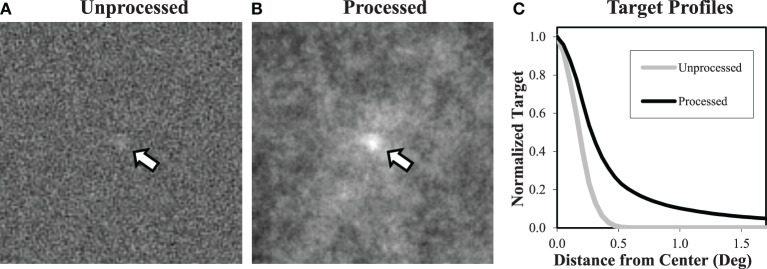
**Processing white noise images**. Processing white noise images **(A)** with the appropriate filter gives them a β = 2 power-law background **(B)**. This transformation also changes the target profile, giving it much longer tails **(C)**. Target contrast (arrows) is enhanced here for display.

In the image processing study, white noise images (i.e., β = 0) were filtered after the target profile was added to have a β = 2 power-law spectrum. The frequency profile of the filter was $\sqrt{S_{2}\left(f\right)}$. As a result of this filtering operation, the target was no longer a Gaussian profile, which reflects the practical reality that image processing alters the properties and appearance of both target and background. Examples of the an image before and after filtering, as well as a plot showing the effect on the target profile, are seen in Figure 3. Detection and localization tasks on the processed images were run as described above.

### The ideal observer

Task efficiency with respect to the ideal observer is the fundamental calculation used in this work. In this section we describe how the ideal observer analysis is implemented, leading to an efficiency estimate.

#### Detection tasks

For the yes-no detection task we identify target-present images as one hypothesis (or class), H_1_, and the target-absent images as the other possible hypothesis, H_0_. We will refer to the images generically as **g**, a column vector of pixel values, with the assumption that the mean background intensity of the stimuli (100 gl in our case) has been subtracted off of the pixel intensities. The Gaussian noise in the images is specified by a multivariate normal distribution (MVN) with a covariance matrix that depends on the power-law exponent of the noise texture, **Σ**β. The conditional distributions of the resulting images are given by

(1) $$\begin{array}{ccc} p\left(g|{\text{H}}_{0}\right) & = & \text{MVN}\left(0,\Sigma_{\beta }\right)\\ p\left(g|{\text{H}}_{1}\right) & = & \text{MVN}\left(s,\Sigma_{\beta }\right). \end{array}$$

Under these conditions, it is well known that the ideal observer can be implemented as a weighted sum of the pixel intensities (Green and Swets, 1966). Let the vector **w**_IO,β_ represent these weights, which are defined in terms of the statistical properties of the images as

(2) $$w_{\text{IO},\beta }=\Sigma_{\beta }^{-1}s.$$

The resulting Ideal observer strategy is implemented by comparing the weighted sum of the image, with mean background subtracted, to a detection threshold,

(3) $$\begin{array}{l} {\text{H}}_{0}:\text{if }w_{\text{IO},\beta }^{T}g<t_{\text{crit}}\\ {\text{H}}_{1}:\text{ if }w_{\text{IO},\beta }^{T}g>t_{\text{crit}}. \end{array}$$

The value of the threshold, *t*_crit_, determines the tradeoff between hits and false alarms. In principle this term should be set on the basis of outcome utilities. However, we leave it as a free parameter to be fit to the human observer data.

With human observer data, we obtain the equivalent contrast for the ideal observer by adjusting contrast and *t*_crit_ until the hit rate and false-alarm rate equal the human observer's. Let *C*^D^_Obs,β_ be the target contrast used for the human observer study, and let *C*^D^_IO,β_ be the equivalent ideal observer contrast. The efficiency of the observer is defined in terms of a squared ratio of contrast thresholds following Kersten (1987) as

(4) $$\eta_{\text{obs},\beta }^{\text{D}}={\left(\frac{C_{\text{IO},\beta }^{\text{D}}}{C_{\text{Obs},\beta }^{\text{D}}}\right)}^{2}\text{​}.$$

Standard errors are determined by calculating efficiency on a session-by-session basis, and then computing the standard error across sessions.

#### Localization tasks

As mentioned in the Introduction, the theory we use for ideal observers in a free-localization task comes from the work of Khurd and Gindi (2005). Here we present a somewhat simplified derivation that is adequate for our purposes. In this case, we have a conditional probability of the data for every possible location of the target. Let **s**_*l*_ represent the profile of the target, when it is centered on the pixel with index *l*, which can be anywhere in the search region (i.e., 128^2^ possible locations). The conditional likelihood of the data given a particular target location is

(5) $$p\left(g|l\right)=\text{MVN}\left(s_{l},\Sigma_{\beta }\right).$$

The basis for localization by the ideal observer is the posterior distribution on possible locations, *p*(*l*|**g**). For a uniform prior distribution on target locations, the posterior distribution is proportional to the likelihood. Under the Gaussian assumptions of our images, we have

(6) $$p\left(l|g\right)=N_{g}{\text{e}}^{s_{l}^{\text{T}}\Sigma_{\beta }^{-\text{1}}g},$$

where *N*_*g*_ is a normalization constant that ensures that *p*(*l*|**g**) sums to 1 over all possible locations.

The task specifies that any response within 5 pixels of the target center is considered a correct response. The ideal observer will therefore choose the location that maximizes the probability of a correct answer. For each point under consideration, the ideal observer adds up the probabilities of all points within a 5-pixel radius, to get a final score for the location. The point with the largest score is then chosen as the ideal observer's response. It is worth noting that the ideal observer response at a given location is very similar to an ideal detector with spatial uncertainty (Pelli, 1985), where uncertainty is confined to the acceptance region around a given location.

The ideal observer decision function can be implemented using convolutions to speed up the computationally intensive steps. For example, the stationary nature of the noise covariance matrix allows the computations of $s_{l}^{T}\Sigma_{\beta }^{-1}g$ to be implemented by convolving the ideal observer template, defined in Equation 2, with the mean-subtracted background. Similarly the computation of the final score at each location in the image can be computed by convolving a disk of radius 5 pixels with the normalized posterior distribution in Equation 6. The recipe for computing the ideal observer begins with pre-computing the ideal observer filter by dividing the Fourier transform of the target by the power-spectrum of the noise. Then for each image, (1) this filter is used in a convolution after the mean background has been subtracted; (2) the result is exponentiated; (3) pixels outside the search region are set to zero; (4) pixels in the search region are scaled so that they sum to 1; (5) the posterior is convolved with a disk of radius 5 pixels; and (6) the maximum point is chosen.

**Figure 4:**
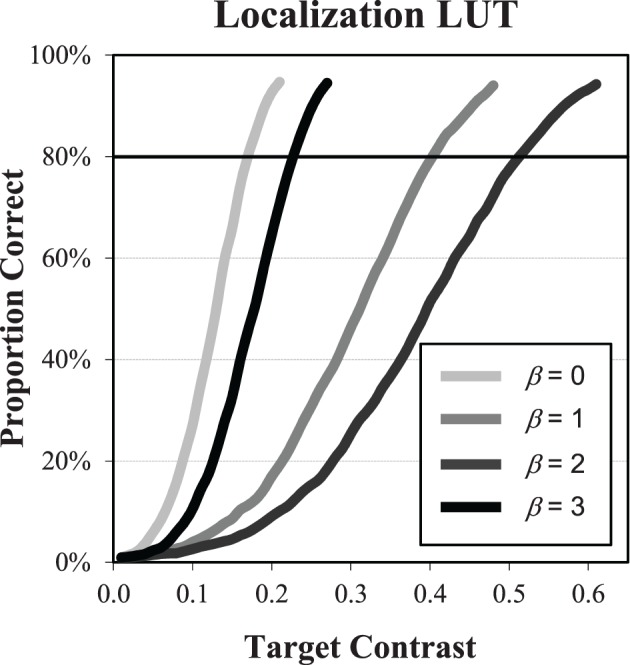
**Ideal observer LUT**. The plots show performance of the ideal observer as a function of target contrast for each of the power-law exponents. These data can be used as a look-up-table for determining the threshold contrast needed by the IO to achieve a given level of performance. For example, at β = 1, the threshold contrast needed to achieve 80% correct is seen to be 0.4.

With a case-by-case ideal observer algorithm, the performance of the ideal observer is estimated to arbitrary accuracy using large sets of sample images. We use this approach to build LUTs of ideal observer performance as a function of target contrast. The LUTs for each β are determined in contrast increments of 0.01 from 0 until PC rises above 94%. The functions are plotted for each β in Figure 4. Each point is based on 2000 sample images, which results in standard errors that are less than 1% near the 80% correct level that is used in the experiments. Inverting these functions allows for us to determine the contrast threshold required by the ideal observer to achieve the specified level of PC. For an observer that achieves a proportion correct of PC_Obs_ in a localization task with a target contrast of *C*^L^_Obs,β_, efficiency is again defined (Kersten, 1987) as the squared contrast ratio

(7) $$\eta_{\text{Obs},\beta }^{\text{L}}={\left(\frac{C_{\text{IO},\beta }^{\text{L}}\left({\text{PC}}_{\text{Obs}}\right)}{C_{\text{Obs},\beta }^{\text{L}}}\right)}^{2}.$$

Standard errors are determined by calculating efficiency on a session-by-session basis, and then computing the standard error across sessions.

The D&L task uses a similar process as the localization task, except that in the last step a threshold is applied. If the maximum score is above the detection threshold, the location of the score is selected for detecting and localizing the target. If the maximum score is below the detection threshold, the ideal observer selects the “target-absent” response. For matching human observer data, the threshold contrast and detection criterion are adjusted to match the rate of correct detect-and-localize responses and the false positive (FP) rate. Efficiency is then calculated as the squared ratio of this contrast to the contrast used in the experiment, as in Equations 4 and 7.

### Classification images

In addition to efficiency, we will use classification images as a way to investigate how visual processing affects task efficiency. This approach is straightforward for the detection tasks, where the classification image analysis has been well developed by Ahumada (2002) and others (Gold et al., 2000; Chauvin et al., 2005; Victor, 2005; Tjan and Nandy, 2006; Murray, 2011). Let **n** represent the noise field for a given trial, with no target profile or mean background. Let us define the quantity **q** as the product of the inverse covariance and the noise field, $q=\Sigma_{\beta }^{-1}n$. The classification image is given by

(8) $$w_{\text{CI}}^{\text{D}}={\bar{q}}_{\text{FP}}-{\bar{q}}_{\text{TN}}+{\bar{q}}_{\text{TP}}-{\bar{q}}_{\text{FN}},$$

where the **q** are the average **q** over the FP, true-negative (TN), true-positive (TP), and false-negative (FN) noise fields. Under the (strong) assumption of a linear template as the mechanism for detecting the target, the classification image will provide an unbiased estimate of the template. If the observer does not follow the linear assumption, the resulting classification image may be distorted, depending on the degree of violation (Ahumada, 2002).

Tjan and Nandy (2006) have analyzed discrimination tasks in the presence of target location uncertainty using classification images. Their approach utilizes the concept of a “clamped signal,” in which the noise field masking the target profile in an incorrect response is analyzed. This approach was found to work well in various two-class detection and discrimination tasks with targets that could be subject to spatial uncertainty. Additionally, Neri (2010) has used early static nonlinearities as a way to model performance in such tasks. In principle, our free-localization task can be considered a classification task with 128^2^ possible response categories (and a somewhat ambiguous definition of a correct response that includes neighboring locations). However, in this work we have pursued a different approach for classification images in which the noise at the location of an incorrect response is used rather than the noise that masked the unchosen target. In this regard, our approach is similar to a previous study by Rajashekar et al. (2006) that used eye-tracking to estimate gaze-contingent classification images, as well as studies that have used the classification-image approach in multiple-alternative forced choice studies (Caspi et al., 2004; Eckstein et al., 2007; Dai and Micheyl, 2010).

Let **n**^*A*^ represent a “response-aligned” noise field, in which the image noise field is shifted so that the location selected by the observer is translated to the center of the image. Let $q^{A}=\Sigma_{\beta }^{-1}n^{A}$, which is analogous to a response-aligned version of **q** defined above. For classification images in the localization tasks, we use the average of the response-aligned **q** vectors when the subject incorrectly localizes (IL) the target

(9) $$w_{\text{CI}}^{\text{L}}={\bar{q}}_{\text{FL}}^{A}.$$

In these cases, the response is entirely driven by the form of the noise at the response location. We will see below that this leads to a strong classification image relative to detection, even though the detection task uses all noise fields in the image and this approach for the free-localization uses approximately 20% of the trials in which a false-localization response is given.

**Figure 5:**
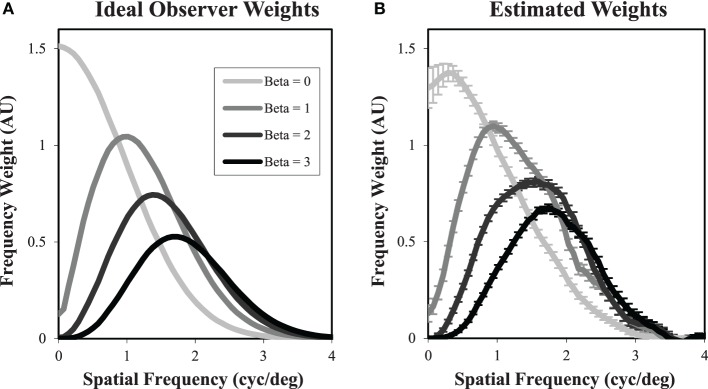
**Classification images in free-localization tasks**. Ideal observer filter weights **(A)** were used to generate responses for each power-law exponent. The filter weights were then estimated from the incorrectly localized noise fields **(B)**. While there is some evidence of bias, particularly for β = 0 at low spatial frequencies, the estimated weights generally give a good sense of the actual filters used to perform the task.

As a simple test of the classification image approach for localization tasks, we have used it to evaluate the ideal observer. Figure 5A shows the frequency weights of the ideal observer, derived analytically from $\Sigma_{\beta }^{-1}s$. In Figure 5B, we see the estimated frequency weights for 2000 trials of the ideal observer using Equation 9, when the target contrast is set so that PC = 80%. While there are some areas of apparent bias, particularly at the lowest spatial frequencies for β = 0, there is generally good agreement between the actual frequency weights used to perform the task and the estimated weights.

## Results and discussion

### Psychometric functions

**Figure 6:**
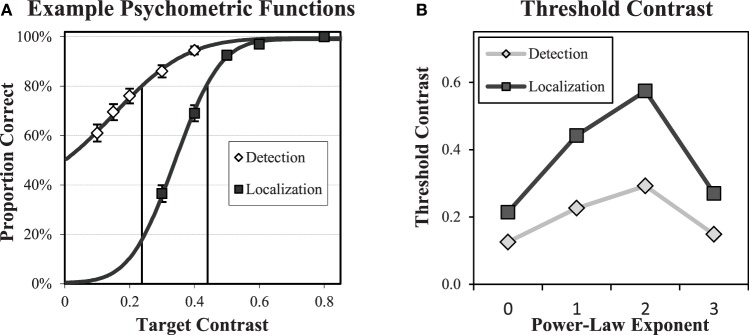
**Psychometric functions and thresholds**. An example of detection and forced-localization psychometric data **(A)** and fitted psychometric functions are shown for one subject in one condition. Error bars = ±1 s.e. The fitting function is a cumulative Gaussian distribution that is used to determine the contrast threshold for 80% correct performance in the subsequent experiments. The average subject contrast thresholds **(B)** in each power-law background is shown for both detection and localization tasks. Standard errors across subjects (not shown) are less than 0.01. The localization tasks requires approximately a factor of 2 greater contrast to obtain equivalent (80% correct) performance.

Contrast thresholds were determined for each subject in each condition from fitted psychometric functions. After an initial training of 5 runs of increasing difficulty totaling 210 trials, psychometric data was acquired in 20 runs of 50 trials at five different contrast levels for a total of 200 trials at each contrast level. The contrast levels used were determined from pilot data. Cumulative Gaussian distribution functions were fit to the proportion of correct responses over the range of contrasts, and contrast thresholds were determined from the contrast that produced 80% correct. An example of the psychometric functions (Subject 4, β = 1) is shown in Figure 6. There was generally good agreement between the subject data and the cumulative Gaussian fitting function.

The average threshold contrast for each task and background type is plotted in Figure 6B. Thresholds within each task peak for b = 2. The thresholds are substantially higher for the localization task, with roughly a factor of two increase for each background.

### Characterizing task performance

After each contrast threshold was determined from the psychometric data, subjects performed a total of 40 runs of 50 trials, for a total of 2000 trials at the subject's threshold contrast. Efficiency with respect to the ideal observer was estimated from this data. The efficiency results are described below in Detect-And-Localize Efficiency. Here, we will describe other measurements that provide additional information to characterize task performance.

**Figure 7:**
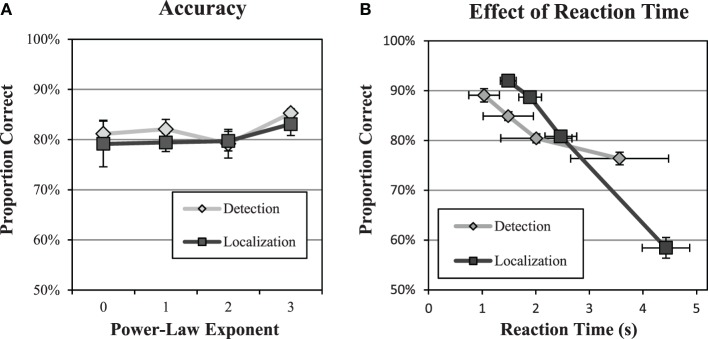
**Accuracy and reaction time**. A check of performance levels in the efficiency data **(A)** shows that performance levels were reasonably close to the targeted 80% level. The midpoint of reaction time in each quartile **(B)** is plotted against performance for the quartile. Averages and standard errors across subjects are shown.

Performance in the efficiency data is reasonably close to the nominal 80% correct levels derived from the psychometric functions. Figure 7A plots average PC across subjects from the efficiency data as a function of the power-law exponent of the background. Overall, PC values averaged 81.9% in the detection experiments and 80.3% in the localization experiments. The slight increases across subjects may be due to learning effects that occurred over the 2000 trials. The largest observed deviation from 80% correct for a single subject in a single condition was 7.3%. These results give us some confidence that efficiency was measured at contrasts near the actual 80% correct threshold.

While reaction time is not an endpoint of our study, this data is recorded as part of the experimental procedure. Reaction time is defined as the time from stimulus onset to the acquisition of a subject response. Median reaction times, given in Table 1, are mostly larger for the free localization task. This is not surprising since the subject need to search an area 6.7 ×6.7° in the localization task. Given the size of this area, the 48% average increase in reaction times seems rather modest. It is worth noting that the increase in median response times is not uniform over the subjects. One subject (S4) is markedly slower in the detection task.

**Table 1:** **Reaction times**.

**Subjects**	**Detection RT**	**Localization RT**	**Rel. dif. (%)**
S1	1.35	2.61	94
S2	1.11	1.80	62
S3	1.17	1.77	51
S4	3.97	2.85	−28
S5	1.01	1.65	64
Ave	1.72	2.13	48

*Median reaction times (RTs) are given for each subject as well as the relative difference between the detection and localization tasks*.

It is also of interest to compare the effect of reaction time and performance as shown in a representative example in Figure 7B. We divided the data into quartiles of 500 trials according to reaction time, and then computed proportion correct in each quartile. The figure plots proportion correct as a function of the median reaction time for the quartile. All subjects exhibited a similar trend of decreased performance with greater reaction times in both tasks. This finding is the opposite of what might be expected from a speed-accuracy tradeoff, where slower speeds allow for more effective task performance. However, decreased performance for longer reaction times has been found previously (Eckstein et al., 2001), and is thought to reflect the effects of a noise limited task where longer reaction times are associated with noise masks that make the task more difficult.

Unlike the detection task, the localization response requires careful positioning of the cursor using the mouse. The accuracy of this process has consequences both for overall accuracy in the task, if mis-positioning the cursor causes the localization response to fall outside the acceptance region, and for aligning the noise fields for the classification image analysis. To get some sense of the accuracy of the localization responses, we have evaluated the deviation of the responses, which is defined as the distance of the subject mouse clicks from the target location for responses that fall within the acceptance region of 5 pixels from the target center. Table 2 gives the average deviation across subjects, in both pixels and degrees of visual angle, and well as the deviation assuming a uniform distribution of responses over the acceptance region. The deviations are all substantially smaller than the uniform distribution would predict, suggesting that there is considerable additional accuracy in the localization response. In addition, there is a consistent decrease in the deviation as β increases. The error represented by the absolute deviation contains both the effects of subject's misperception of the target center, as well as motor noise in the subject's response. Of these two, motor noise will be detrimental to the classification image methodology, since it will lead to misalignment of the selected noise fields. The observed deviations in Table 2 act as an upper bound on motor noise in the subject responses, and suggest that these effects may be modest.

**Table 2:** **Localization accuracy**.

**Abs. dev**.	**β = 0**	**β = 1**	**β = 2**	**β = 3**	**Uniform**
Pixels	1.96 ± 0.08	1.69 ± 0.11	1.52 ± 0.14	1.41 ± 0.15	3.40
Degrees	0.102 ± 0.004	0.088 ± 0.006	0.079 ± 0.007	0.074 ± 0.008	0.180

*Average absolute deviation (±s.e. across subjects) of correct localization responses relative to the target center. Data is given both in pixel units as well as degrees of visual angle. For reference, the absolute deviation assuming a uniform distribution within the acceptance region is also given*.

### Task efficiency

**Figure 8:**
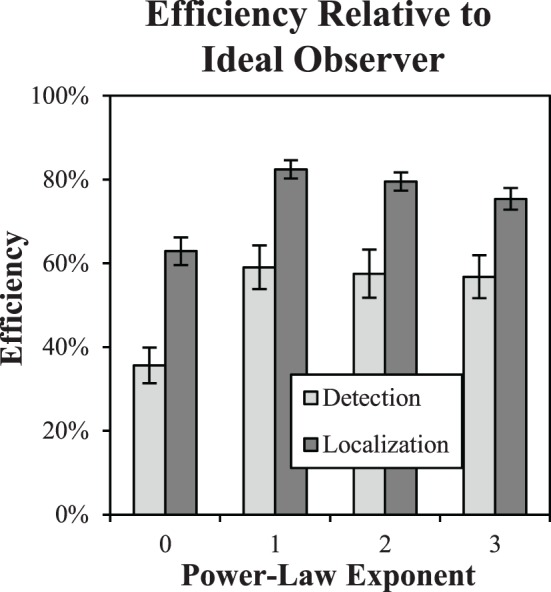
**Task efficiency**. Efficiency of detection and localization tasks is plotted as a function of the power-law exponent, showing a substantial increase for localization tasks. Error bars are ±1 s.e.

The primary performance result we are interested in for these studies is observer efficiency, as plotted in Figure 8. Efficiency with respect to the ideal observer appears to be substantially higher for localization tasks than detection tasks. A Two-Way ANOVA with the five subjects considered as replications finds significant effects for both the task [*F*_(1, 32)_ = 63.4, *p* < 0.0001] as well as the background exponent [*F*_(3, 32)_ = 11.7, *p* < 0.0001]. The interaction between task and exponent was not found to be significant [*F*_(3, 32)_ = 0.39, *p* > 0.76]. It should be noted that average efficiency near 80% for β-values of 1–3, is considered quite high. In the classic experiments by Burgess et al. (1981), efficiency as high as 70% was observed with averages across observers closer to 50%. These experiments used a spatial forced choice methodology and white noise (β = 0). Experiments in low-pass noise similar to the β = 3 condition used here (Abbey and Eckstein, 2007), found efficiency in the 40 to 60% range. These are consistent with our findings in the detection task, all of which utilize aperiodic “bump” targets. Efficiency of oscillatory targets are typically lower (Legge et al., 1987). The increased efficiency we find in the localization tasks represents a substantial gain from these fixed-location tasks, and suggests that subjects have little room for sub-optimal computations in performing these tasks.

Efficiency is somewhat lower for β = 0 in both the detection and localization tasks. We consider this case further in Efficiency of Image Processing for β = 0. below. We also note that these efficiency values appear to be relatively stable with the acceptance radius. We observed less than a 1% difference in observed efficiency varying the acceptance region from 4 pixels to 7.

These efficiency results show that in spite of larger thresholds for the free-localization relative to detection, as shown in Figure 6B, overall efficiency is substantially higher. This means that thresholds for the ideal observer increase proportionally even more than the human subjects' did. Our findings are consistent with the uncertainty hypothesis (Tanner, 1961; Pelli, 1985), which posits imperfect use of the location cues in detection tasks, and leave the observer with some residual uncertainty regarding the location of the target that can reduce performance. The ideal observer is not subject to this phenomenon, which results in a somewhat lower contrast threshold. In the free-localization task, where uncertainty is intrinsic to the task, the ideal observer does not have the advantage of precise knowledge of location, and contrast thresholds rise relative to the human observers as a result. However, other explanations for the large difference in efficiency are possible. For example, detection tasks require that the subject use some sort of criterion that dichotomizes responses. If this criterion drifts or is prone to jitter, performance will be reduced. This possibility motivated the detect-and-localize study.

### Detect-and-localize efficiency

**Figure 9:**
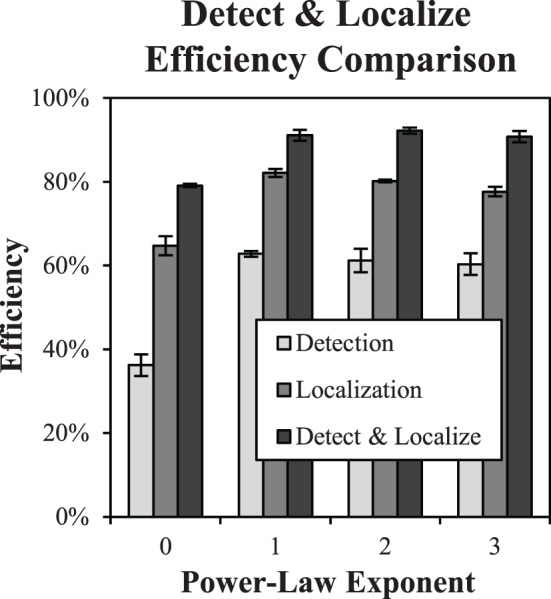
**Detect and localize efficiency**. The plot shows detect-and-localize efficiency compared to detection efficiency and localization efficiency for each power-law background. Error bars are ±1 s.e. Small differences with Figure 8 (detection efficiency and localization efficiency) are due to limiting the averages to the three subjects that participated in the D&L study.

A subset of three subjects performed the detect-and-localize experiments, which were all run after the detection and localization data were acquired. Threshold target contrast from the localization tasks were used as target contrasts for these experiments. The proportion of correct responses dropped modestly from an average of 80.3% in the localization tasks to 76% in the D&L tasks. Figure 9 plots shows the efficiency data for the detection task, localization task, and D&L tasks as a function of the power-law exponent for the subset of subjects that participated in all three studies. The average efficiency values for the D&L are all well above both the detection and localization tasks. In fact several observed values are near 90% efficiency, which is again quite high for tasks masked by luminance noise. These findings are close to the highest reported efficiency we are aware of for visual tasks limited by noise (Manjeshwar and Wilson, 2001).

### Efficiency of image processing for β = 0

Figure 8 shows reduced efficiency in the β = 0 condition of both the detection and localization tasks. After finding this effect, we were interested in whether it might be mitigated by processing the images to have a background power-law of β = 2, where efficiency was generally better. As described above in Stimulus and Display Properties, this is accomplished by filtering the images with a kernel that has a 1/*f* spectrum, which will modify both the background statistics and the target profile, as shown in Figure 3.

**Figure 10:**
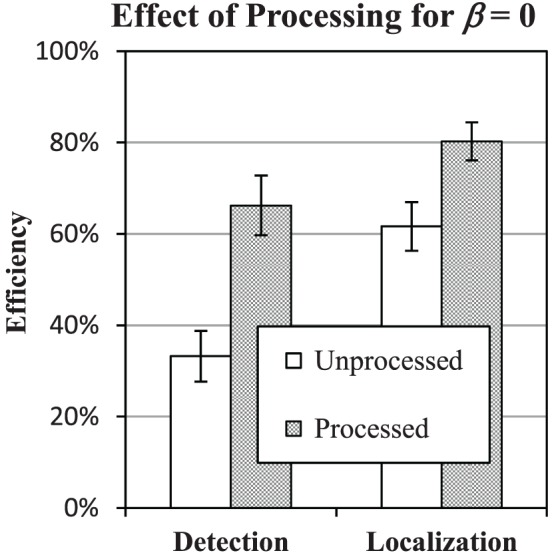
**Effect of processing the β = 0 condition**. Efficiency of detection and localization tasks in β = 0 condition is plotted against efficiency with (processed) and without (unprocessed) filtering the images to have power-law spectrum with β = 2. Error bars represent ±1 s.e. Small difference between the unprocessed data and Figures 8, 9 are due to limiting the averages to the three subjects that participated in the processing study.

Figure 10 shows that the effect of processing is to bring efficiency in the β = 0 condition up to 66% in the detection task and 80% in the localization task. These levels are consistent with efficiency levels found for β in the range of 1–3.

### Classification images

**Figure 11:**
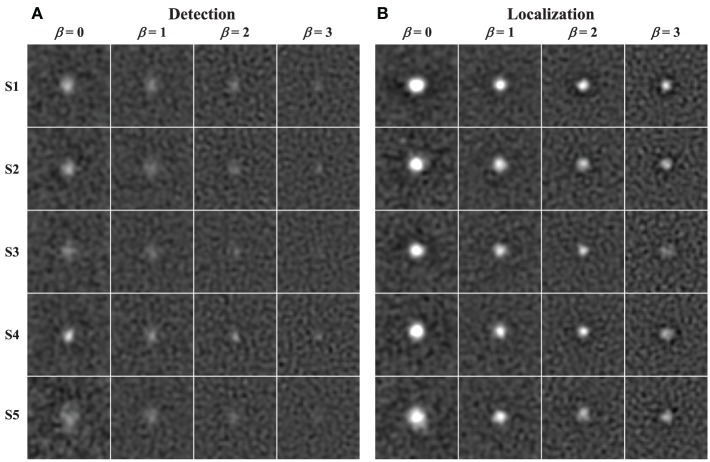
**Classification images**. Estimated classification images (cropped to 2.1° per side) are shown for each condition (columns) and subject (rows) in the detection **(A)** and localization **(B)** tasks. The images are windowed to have approximately the same magnitude of estimation error.

Figure 11 shows the classification images for each subject in each background condition for both the detection and localization tasks. The images are cropped to the central 2.1° of visual angle (40 pixels). Outside of this area, there are no discernable features beyond what appears to be estimation error in the classification images. To mitigate the effects of noise, the classification images have been low-pass filtered with a 4th-order Butterworth filter, with the roll-off parameter set to 5.6 cyc/deg (0.29 cyc/pixel). This was well beyond the point at which the spatial frequency plots below appear to decay to zero.

The images in Figure 11 were windowed to have approximately the same mean background and error magnitude. Thus, the intensity of the features in the observed classification images gives some sense of their signal-to-noise ratio (SNR). The generally brighter appearance of classification images in the localization tasks relative to the corresponding detection tasks suggests that search process may lead to methodological advantages for estimating classification images, even though the localization classification images are estimated from approximately 20% of the subjects responses in which an incorrect localization response if given. There also appears to be some differences in the intensity of the classification images going from β = 0 to β = 3, and there are clearly individual differences between subjects.

**Figure 12:**
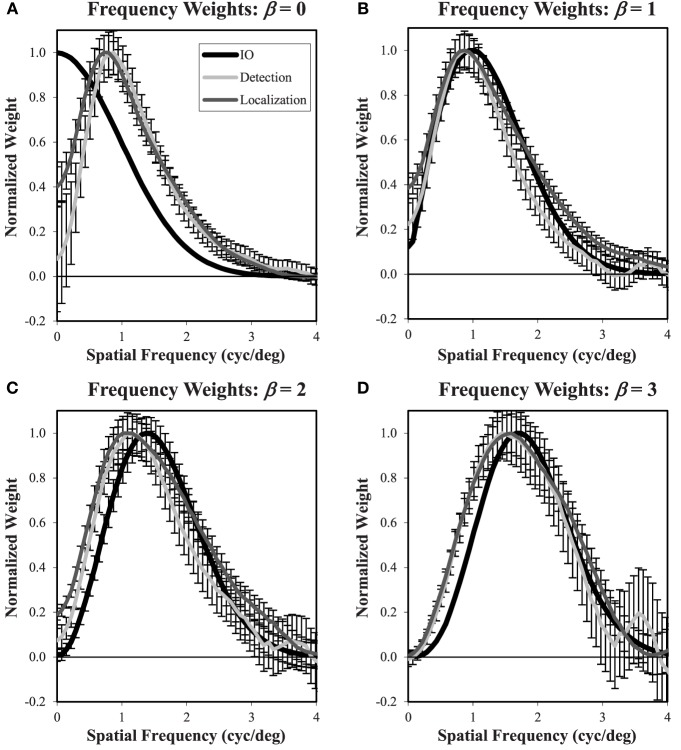
**Frequency weights derived from Classification images**. Radial frequency profiles are shown for each of the four power-law textures **(A–D)** with normalization so that the maximum weight is one. The ideal observer profile is derived from theory. The detection and localization plots are averaged across the five subjects. Error bars are ±1 s.e. averaged across subjects. The legend **(A)** applies to all plots.

In addition to the overall intensity of the classification images, we are also interested in the profile of these decision weights. Based on previous experience, we find that differences between classification images in different conditions are most clearly depicted for radial averages in the spatial-frequency domain. Figure 12 plots the classification frequency weights averaged over subjects and normalized so that the weight at the peak frequency is 1. To reduce the effects of noise in the classification images, a Butterworth spatial window with a cutoff of 1.05° (20 pixels) was applied before the Fourier transform and radial averaging. For reference, we have plotted the classification weights of the ideal observer as well. In all conditions, the average frequency weights assume a bandpass form, peaking at frequencies between 0.7 cyc/deg and 1.6 cyc/deg as β goes from 0 to 3. As has been found previously (Abbey and Eckstein, 2007; Conrey and Gold, 2009), the classification weights here give evidence of visual processing that is changing with the different power-law textures in the background. But this process is not as extreme as the adaptation that occurs in the ideal observer, where peak frequencies move from 0 to 1.7 cyc/deg.

In the β = 0 condition, we observe substantial underweighting of low spatial frequencies relative to the ideal observer. Of interest for the comparison of detection and localization tasks, there is less low-frequency suppression in the localization task compared to the detection task. As β increases, we see that the low-frequency profiles come together, but now they do not suppress low frequencies as much as the ideal observer. Also, as β increases, the classification weight frequency profiles begin to diverge at higher spatial frequencies above the peak values. Here the profiles from the localization tasks have higher weights that are closer to the ideal observer.

**Figure 13:**
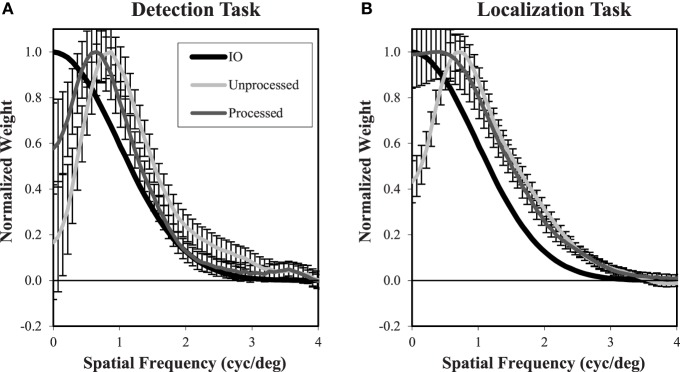
**Frequency weights for processed and unprocessed images**. These plots are similar to Figure 12 and show estimated weights from the β = 0 images using the responses to processed and unprocessed images. In both the detection **(A)** and localization **(B)** tasks, the effect of image processing is to increase the estimated weights at low spatial frequency, bringing them closer to the ideal observer weights. The legend **(A)** applies to both plots.

Figure 13 shows the frequency plots in the β = 0 condition using responses from the processed and unprocessed data averaged over the three subjects that participated in these studies. The plots show processing effectively modifies the weighting profile that subjects use. In both tasks, the effect of processing is to increase the low-frequency weighting so that the average subject classification weights more closely match the ideal observer. Thus, the classification image profiles give a visual mechanism for the improved efficiency found in Figure 10.

## Summary and conclusions

We find human observers substantially improve in performance relative to the ideal observer in free-localization tasks compared to fixed-location detection tasks, in spite of increased contrast thresholds. This occurs in all four power-law textures that were investigated. In a follow-up study investigating a detect-and-localize task, we find the highest measured efficiency in our experiments, suggesting that our efficiency results are not simply a consequence of a general inability to maintain detection criteria. Our findings are consistent with spatial uncertainty as a limiting effect in the presence of location cues.

While it is clear from the classification images that observers are able to tune their visual templates to the statistics of the noise in the images, there is also evidence that this process is limited in both fixed and free-localization tasks. Despite a common target profile, the different power-law textures require different frequency tuning to achieve optimal performance. We do find some evidence of such tuning in the classification images estimated from the subject responses. Peak spatial frequency weights change by roughly a factor of two going from a power-law exponent of β = 0 to β = 3 (0.72–1.59 cyc/deg). However, on average the subject frequency weights exhibited some clear departures from optimal tuning as defined by the ideal observer. At β = 0, we find human observer frequency weights shifted to higher spatial frequencies relative to the ideal observer. For β > 0, human-observer classification weights peak at lower spatial frequencies than the ideal observer.

Frequency tuning of subjects in the white-noise condition was most different from the ideal observer. This condition also led to the lowest efficiency in performance. Since β = 0 was the power-law exponent furthest from that found in natural scenes (β = 2), this finding is consistent with the idea that the human visual system is somewhat adapted to the statistics of natural images. The follow-up study investigating processed images supports this connection by finding uniformly improved performance when the white-noise images were filtered to have β = 2. Filtering the images was also seen to effectively improve frequency tuning of the subjects in the white-noise condition.

While we do not attempt to explicitly model the visual system to explain our findings, we do believe that our findings may be relevant in such attempts, for the same reasons given originally by Burgess (Burgess et al., 1981). The finding of high efficiency in free-localization and detect-and-localize tasks suggest that models of vision in these tasks cannot be very different, at a computational level, from the ideal observer, and thus may provide a valuable constraint to such efforts in future studies.

### Conflict of interest statement

The authors declare that the research was conducted in the absence of any commercial or financial relationships that could be construed as a potential conflict of interest.
